# Perceptual-Semantic Congruency Facilitates Semantic Discrimination of Thermal Qualities

**DOI:** 10.3389/fpsyg.2017.02113

**Published:** 2017-12-06

**Authors:** Yizhen Zhou, Hsin-Ni Ho, Junji Watanabe

**Affiliations:** ^1^Department of Information and Communications Engineering, Tokyo Institute of Technology, Tokyo, Japan; ^2^NTT Communication Science Laboratories, Nippon Telegraph and Telephone Corporation, Kanagawa, Japan

**Keywords:** thermal-semantic interactions, thermal perception, semantic processing, perceptual-semantic congruency, embodiment

## Abstract

The ability to sense temperature is vital to our life. It signals the environmental condition, reflects the physiological conditions of our own body, and generates feelings of pleasantness or unpleasantness. Moreover, recent studies have demonstrated implicit associations between physical temperature and social/emotional concepts, suggesting the processing of temperature may even influence cognition. In this work, we examined the effect of physical warmth and coldness on semantic cognition. Participants performed speeded target categorization for thermal descriptors in the form of semantic words or illustrative figures representing the thermal qualities “warm” or “cold” while physical thermal stimulation was presented. We compared the average reaction time (RT) for the congruent and incongruent conditions managed by response key assignments. In the congruent condition, the response key for the symbol associated with warmth (coldness) was assigned to the hand with warm (cold) thermal stimulation, and in the incongruent condition the key assignment was reversed. Our results demonstrate that the average RT in the congruent condition was faster than in the incongruent one for both forms of thermal descriptors, suggesting that the experience of physical temperature facilitates the internal processing of the meaning of thermal quality.

## Introduction

The ability to sense temperature is vital to our life. It not only reflects the physiological conditions of our own body but also plays an important role in manual exploration and object recognition. By directly touching an object, the feeling of coldness or warmness can give us a clue to its material composition, such as whether it is made from metal or wood (Ho and Jones, 2006). Together with other haptic cues, thermal cues provide the most fundamental and direct means of contact with the environment, and they might also integrate other perceptual cues and thus enhance our understanding of it (Kanaya et al., 2012; Ho et al., 2014a).

More interestingly, studies have revealed associations between temperature stimulation and cognition. In recent years, a rapidly growing body of research on “embodied cognition” as a conceptual framework for understanding the mind has suggested that central representations in cognition do not exist independently of the sensory-motor systems, but are rather grounded in the environment, body states, and stimulations (Barsalou, 2008). Therefore, higher levels of human information processing, such as semantic processing, may be influenced by and associated with bodily perceptions. In fact, empirical demonstrations have shown associations between physical temperature stimulation and cognition. For instance, both our social judgment of other people in terms of the personality trait “warmth” and how we treat others are influenced by temperature stimulation in experiments on human behavior at the interpersonal level (Williams and Bargh, 2008). Inagaki and Eisenberger (2013) showed that people feel more “connected” after holding a warm object and warmer after reading positive messages; and feelings of belonging were found to be threatened when people with low family support drink cold water (Chen et al., 2014). Moreover, the experience of cold temperatures even improves the performance of an anti-saccade task, as “cold behavior” is a concept used to describe rational and deliberate behavior (Halali et al., 2017).

Although studies have demonstrated associations between thermal sensory processing and cognition, few have investigated the interaction between thermal and semantic processing. Semantics research is concerned with the process of acquiring meaning from semantic representations (symbols). Classical amodal theories argue that concepts and meaning are generated by the combination of arbitrary symbols which have little connection with the external world and are independent of perception and action (Fodor, 1975, 1983, 1998; Landauer and Dumais, 1997; Foltz et al., 1998; Pylyshyn, 1999). However, recent alternative theories, i.e., the “embodied” models, suggest that the internal representation is implemented via stimulations in the brain's modal systems and interfaced with the external structures (Barsalou, 2010). Specifically regarding temperature stimulation, one study revealed an implicit association between emotional and thermal concepts with semantic and physical thermal stimulation (Bergman et al., 2014), but whether there is a direct link between thermal processing and its corresponding semantic processing has not been investigated.

In this study that is motivated by the embodied cognition theory, we performed psychophysical experiments to gain more solid evidence of the physical and semantic interaction in thermal quality and determine the strength of the association between physical temperature and semantic processing. We used speeded target discrimination tasks with symbols expressing the thermal qualities “warm” and “cold” while presenting physical thermal stimulation to the participants' hands. Target stimuli divided into two groups of meaning, “warmth” or “coldness”, were shown in the form of semantic words (Experiment 1) and illustrations (Experiment 2). The congruent and incongruent conditions were managed by response key assignments. The reaction time (RT) was the main parameter for evaluating the performance of the discrimination tasks. Our results demonstrate that the average RTs in the congruent condition (i.e., words contain meaning of warmth assigned to the hand with a warm thermal sensation) were faster than in the incongruent one (i.e., words contain meaning of warmth assigned to the hand with a cold thermal sensation). Our results indicate that physical-semantic congruency in thermal quality can facilitate the semantic processing of symbols.

## Materials and methods

### Participants

Ten paid subjects (mean = 32.7 years; *SD* = 9.21; six females) participated in the semantic word experiment (Experiment 1). Twelve paid subjects (*M* = 33.8 years; *SD* = 9.06; eight females), including the 10 who took part in Experiment 1, participated in the illustration experiment (Experiment 2). All the participants had normal or corrected-to-normal vision and normal motor and linguistic functions. The experiment was approved by the ethics committee of NTT Communication Science Laboratories and conducted in accordance with the ethical standards in the 2013 Declaration of Helsinki. Written informed consent was obtained from all participants in advance.

### Apparatus

**Figure 1:**
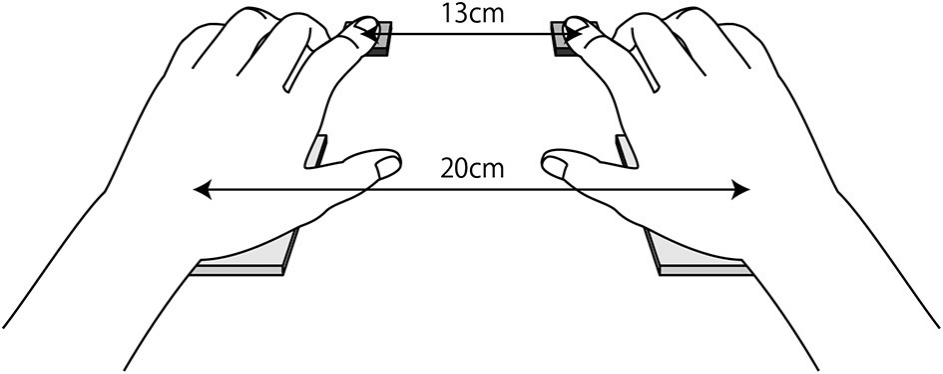
Response keys and thermal display.

The experimental system comprises a set of thermal displays, a liquid crystal display (LCD, Model: L761T-C, EIZO Corporation), and numeric keyboards (Model: SANWA SUPPLY NT-19UH2BK), and it was designed for this study. The thermal display consists of two Peltier devices (TEC1-12730, Hebei I.T. Co., Ltd) with a touchable surface area of 62 × 62 mm, with two electric fans placed underneath them. The temperature of the Peltier devices was controlled by computer (Windows XP), and a proportional integral-differential (PID) controller programmed in MATLAB monitored the surface temperature of the Peltier devices and maintained it at the desired one. The experimental trials were programed by MATLAB, and responses were made via two response keys on two numeric keyboards. RTs were measured using the built-in functions of Psychtoolbox. Participants pushed response keys using the index finger with the palm on the thermal display as shown in Figure 1. The distances between the response keys and between the Peltier devices were 13 and 20 cm, respectively. The participants were seated in front of a 19.6-inch LCD computer monitor with a resolution of 1,280 × 1,024 pixels and a refresh rate of 60 Hz. The distance between the participant's eyes and the screen was ~50 cm. The experiment was conducted in a room with air-conditioning to keep the room temperature at 28°C.

### Stimuli

**Figure 2:**
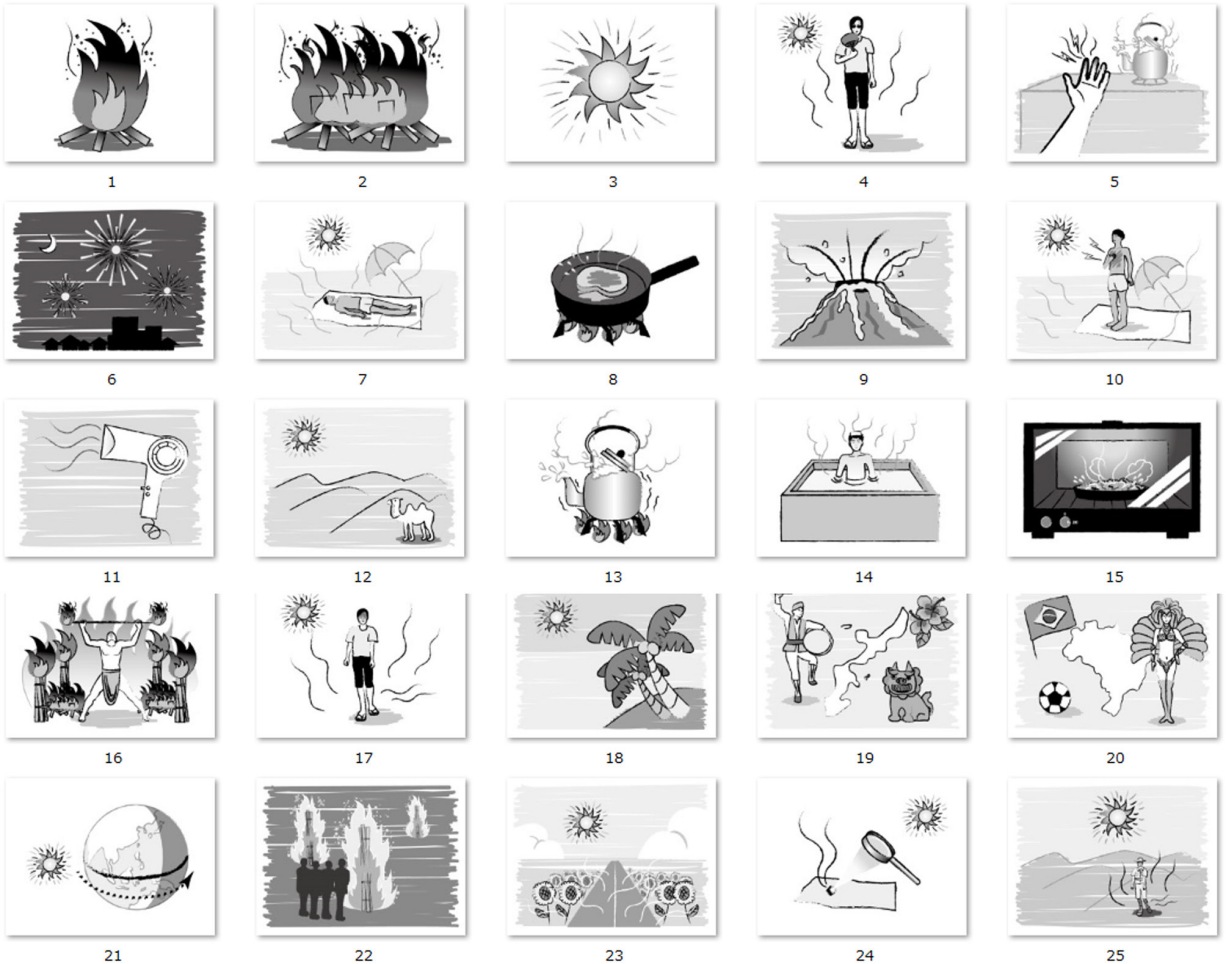
Full list of illustrations associated with meaning of warmth.

**Figure 3:**
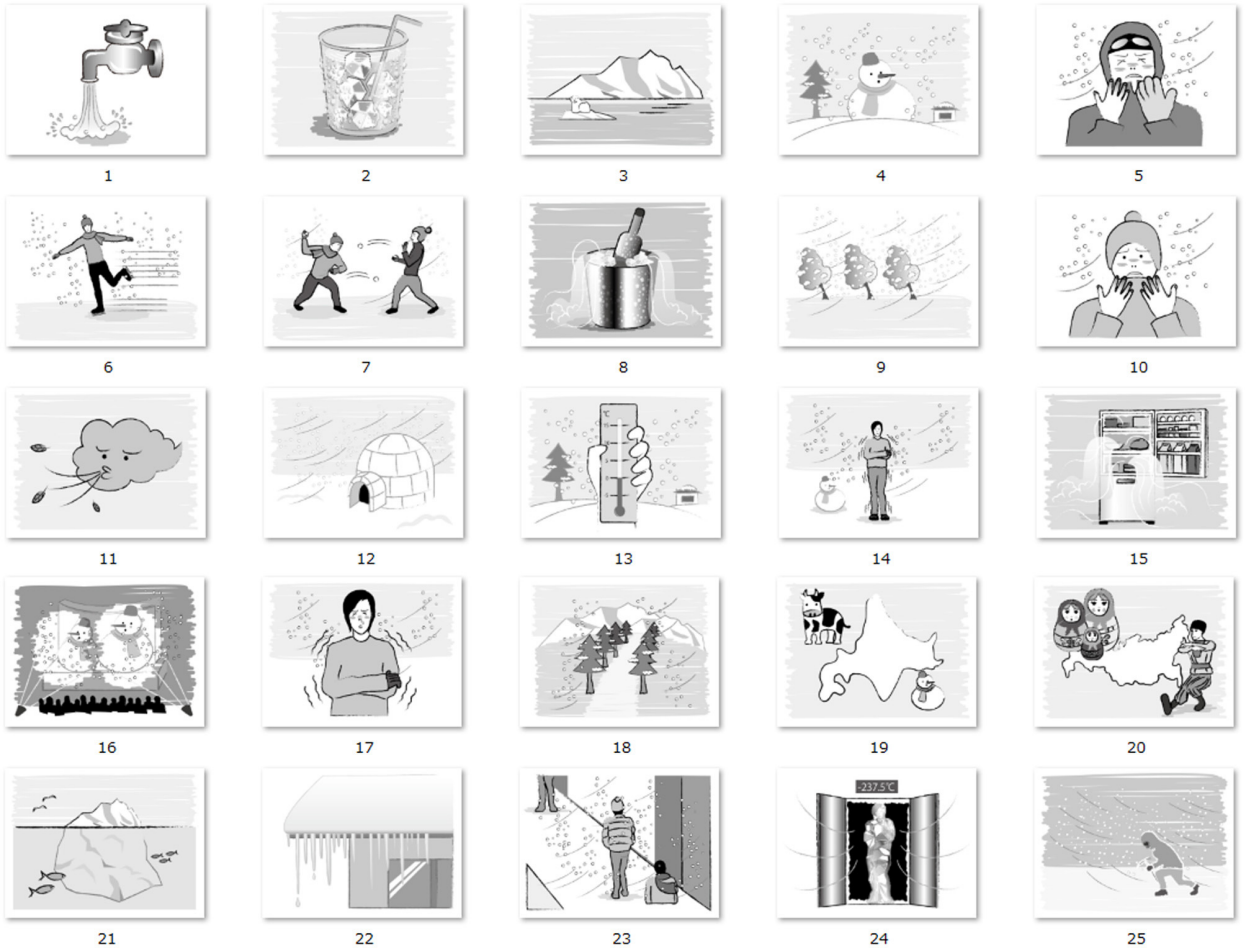
Full list of illustrations associated with meaning of coldness.

In Experiment 1, 50 words were used in total, and they were divided into two categories (25 for warmth and 25 for coldness, see Tables 1, 2). For the selection of the 50 words, we made a list of frequent used Japanese words associated with warmth and coldness, and five native Japanese speakers (one author and four naïve native Japanese speakers who did not participate the Experiment 1 and 2) selected the words. The target words were in Japanese because the native language of all participants was Japanese. The average length of warm/cold words were 2.44 and 2.4 characters, respectively. There were 22 nouns, two adjectives, and one verb in each category (warm or cold). The thermal words were presented in white with 180 pt Arial font (~45 × 45 mm for each character) on a black background. In Experiment 2, we used fifty illustrations with meanings identical to the words in Experiment 1 (see full list in Figures 2, 3). The size of each image was 800 × 600 pixels. To exclude the effect of color, the images contained no color information.

**Table 1:** List of words associated with meaning of warmth.

**No**.	**Words**	**Translation**
**WARMTH**		
1	火	Fire
2	炎	Flame
3	太陽	Sun
4	夏	Summer
5	火傷	Burn
6	花火	Fireworks
7	日光浴	Sunbathing
8	焼く	Bake
9	マグマ	Magma
10	日焼け	Sunburn
11	熱風	Hot air
12	砂漠	Desert
13	沸点	Boiling point
14	暖かい	Warm
15	オーブン	Oven
16	火祭り	Fire festival
17	熱中症	Heatstroke
18	熱帯	Tropics
19	沖縄	Okinawa
20	ブラジル	Brazil
21	赤道	Equator
22	火柱	Pillar of fire
23	真夏日	Hot summer day
24	発火点	Ignition
25	灼熱	Scorching heat

**Table 2:** List of words associated with meaning of coldness.

**No**.	**Words**	**Translation**
**COLDNESS**		
1	水	Water
2	氷	Ice
3	北極	North pole
4	冬	Winter
5	凍傷	Frostbite
6	スケート	Snow skate
7	雪合戦	Snowball fight
8	冷やす	Cool
9	吹雪	Snowstorm
10	しもやけ	Chilblains
11	北風	North wind
12	凍土	Frozen ground
13	氷点	Freezing point
14	寒い	Cold (feeling)
15	冷蔵庫	Refrigerator
16	雪祭り	Snow festival
17	風邪	Cold (illness)
18	寒帯	Frigid zone
19	北海道	Hokkaido
20	ロシア	Russia
21	氷山	Iceberg
22	氷柱	Icicle
23	真冬日	A day temperature below °C
24	絶対零度	Absolute zero
25	極寒	Midwinter

To confirm the appropriateness of the words and images in terms of the association with warm/cold concepts, participants in Experiment 2 evaluated the subjective thermal intensity of each word and image (images were rated before the experiment, separately with words the original ratings can be found in Data sheet in Supplementary Material). The subjective ratings were obtained with a 7-point scale from −3 (extremely warm) to 3 (extremely cold) for “cold” descriptors and from −3 (extremely cold) to 3 (extremely warm) for warm descriptors. The group mean of subjective ratings was 2.24 for “cold” ones and 2.49 for “warm” ones for words; and 2.32 for “cold” ones and 2.35 for “warm” ones for images. These values correspond to the labels between “extremely cold/warm” and “a bit cold/warm” for cold and warm descriptors, respectively, indicating our selection of words and illustrations shares a common conception of warmth/coldness, at least in the realm of Japanese language.

For the physical stimulation of temperature, we manipulated the skin temperature of participants' hands rather than the room temperature, as the changes in skin temperature may be important to influence the semantic processing. Previous studies suggest local warming/cooling at skin can influence emotional and social behavior (Williams and Bargh, 2008; Bergman et al., 2014); and even the social concepts can be reflected in the changes in skin temperature (Ijzerman et al., 2012). The physical stimulations for warm and cold were set at 37 and 27°C, respectively. The temperatures were chosen for their similarity in terms of subjective thermal intensity and for the innocuous thermal sensations they produce (see Greenspan et al., 2003). Because the neutral temperature of human skin is 32°C, the 5°C difference was enough to induce a warm or cold thermal sensation, and participants confirmed that they were able to feel the warm or cold temperature on both palms before the start of experiment.

### Procedure

We performed the speeded target discrimination tasks using semantic words in Experiment 1. Participants experienced the physical temperature by putting their palms on the Peltier devices' surfaces. They were asked to memorize the instructions shown on the screen at the beginning of every experimental session and press corresponding keys to discriminate warmth and coldness as quickly and accurately as possible once they has seen the words that appeared on the screen. Before each experimental session, participants confirmed whether they felt “cold” and “warm” on each palm. The distance between a key and Peltier device was adjusted according to the size of participants' hands, and once the set position was fixed, the participants were instructed not to move the palm on the surface of Peltier device during the experiment.

The experiment included twelve sessions divided into two blocks (six sessions in each block). Each session had 50 trials (25 trials for each thermal quality). Each trial began with the presentation of a fixation point in the center of the screen for an interval of 800 ms. The target stimulus was then presented centrally until the participant responded. Experimental trials were presented in a different random order for each participant.

Between each session, there was a 3-min adaptation period during which participants placed their palm onto a silicon rubber plate heated to 32°C to adjust their skin temperature to a neutral one to ensure they would perceive the desired thermal sensation. There was a 30-min break between blocks. The first two sessions in each block were practice sessions, in which the neutral thermal stimulation at 32°C was presented to both hands, with the response key for the warm meaning assigned to either the left or right hand and the one for cold meaning assigned to either the right or left hand. The response key assignment was reversed between the two sessions. The third and fourth (or fifth and sixth) sessions were congruent conditions and the fifth and sixth (or third and fourth) sessions were incongruent ones. In the congruent sessions, the warm and cold stimuli were presented to the right and left (or left and right) hands, with the response key for the warm meaning assigned to the hand with the warm stimulation and the one for the cold meaning assigned to the hand with the cold stimulation. In the incongruent sessions, the warm and cold stimuli were presented to the right and left (or left and right) hands, with the response key for the warm meaning assigned to the hand with the cold stimulation and the one for the cold meaning assigned to the hand with the warm stimulation. The location of the thermal stimulation was reversed in two sessions of the same conditions. In summary, an experimental block included two practice sessions, two congruent condition sessions, and two incongruent condition sessions. Two experimental blocks were performed. The order in which a condition (congruent and incongruent) was presented first in the blocks was counterbalanced across participants. Each block took around 30 min per person to complete. The participants' RTs and response accuracy were collected.

The procedure in the illustration experiment (Experiment 2) was exactly the same as in Experiment 1, but with images as target stimuli displayed on the screen. At the beginning of the illustration experiment, the participants rated the illustrations regarding the subjective thermal intensity of each image (pre-experiment rating). For the rating, they used two types of 7-point scales for counterbalancing the polarization of scaling: extremely cold, cold, a bit cold, none, a bit warm, warm, extremely warm; and extremely warm, warm, a bit cold, none, a bit cold, cold, extremely cold. The ratings were converted into numeric representations and analyzed later.

The pre-experiment rating process also helped participants learn and memorize the meanings of each image represented. The order of showing the “cold-meaning” illustrations or “warm-meaning” ones first for the subjective rating was also counterbalanced across all the participants. In addition, the participants also rated the subjective thermal intensity they perceived from the words we selected in Experiment 1 using the same rating scales.

### Data analysis

The results of congruent and incongruent trials (4,000 trials) for all ten participants in Experiment 1 were analyzed. There are 6,000 trials in total including the practice trials using neutral thermal stimulation. Due to a failure of acquisition from key pressing, 1.10% of the data (66 trials) were invalid, and 2.14% of the responses (127 trials) in the trials after the failure were incorrect. Hence, they had to be excluded.

For Experiment 2, the results of congruent and incongruent trials (4,800 trials) for all 12 participants were analyzed. There are 7,200 trials in total including the practice trials using neutral thermal stimulation. Due to a failure of acquisition from key pressing, 1.07% of the data (77 trials) were invalid, and 2.92% of the responses (208 trials) in the trials after the failure were incorrect. Hence, they had to be excluded.

## Results

### Experiment 1: effect of physical warmth and coldness on semantic words

**Figure 4:**
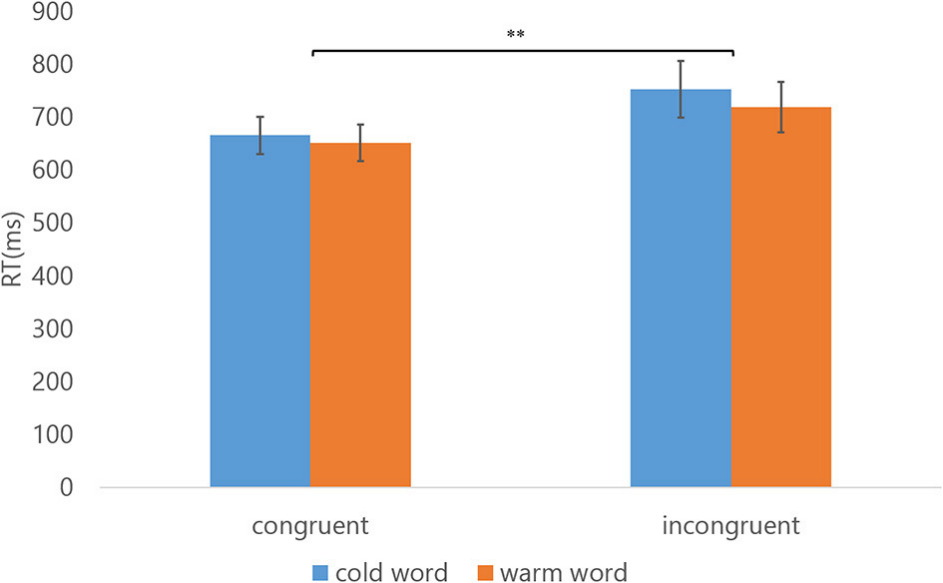
Results indicating the mean of RTs (Experiment 1). The target stimuli in two categories are indicated by the color of the bars, with blue and orange bars representing cold word and warm words, respectively. The error bars show the standard error of the mean. ^**^Indicate statistical significance of *p* < 0.01 (three-way repeated measures ANOVA).

We obtained RTs for each condition from an experimental block of a participant and averaged them. A three-way repeated measures ANOVA was conducted using SPSS Statistics software with congruency effect (congruent/incongruent combinations), meaning of words (“warm” and “cold” words), and the test order of conditions in a block (congruent first/incongruent first) as factors and the RT as the dependent variable. The RTs averaged across participants are shown in Figure 4. The main effect of congruency was significant [*F*_(1, 9)_ = 18.246, *p* = 0.002, η^2^ = 0.670]. The RTs for the congruent condition were shorter than those for the incongruent condition. The difference in RTs for cold words in congruent and incongruent conditions was 88 ms (666 vs. 754 ms); for warm words it was 69 ms (651 vs. 720 ms). The main effect of meaning was not significant, [*F*_(1, 9)_ = 4.147, *p* = 0.072, η^2^ = 0.315], although the average RTs for warm words was somehow faster than for cold ones. There was no significant effect of test order [*F*_(1, 9)_ = 0.022, *p* = 0.886, η^2^ = 0.002]. In other words, whether the congruent condition or incongruent condition was first in a block did not affect the RTs. We expected no interaction between meaning and congruency because word lengths o and word classes were similar in the warm or cold words, and this is consistent with the fact that no significant interactions were observed, [for meaning × congruency, *F*_(1, 9)_ = 1.497, *p* = 0.252, η^2^ = 0.143]; [for congruency × test order, *F*_(1, 9)_ = 0.277, *p* = 0.611, η^2^ = 0.030]; [for meaning × test order, *F*_(1, 9)_ = 0.653, *p* = 0.440, η^2^ = 0.068]; [for meaning × congruency × test order, *F*_(1, 9)_ = 0.432, *p* = 0.528, η^2^ = 0.046]. The estimated Bayes factor for congruency was significantly large, [*BF*_10_ = 1326000], which indicates the current sample size was sufficient for obtaining very strong evidence of the congruency effect.

### Experiment 2: effect of physical warmth and coldness on illustrations

**Figure 5:**
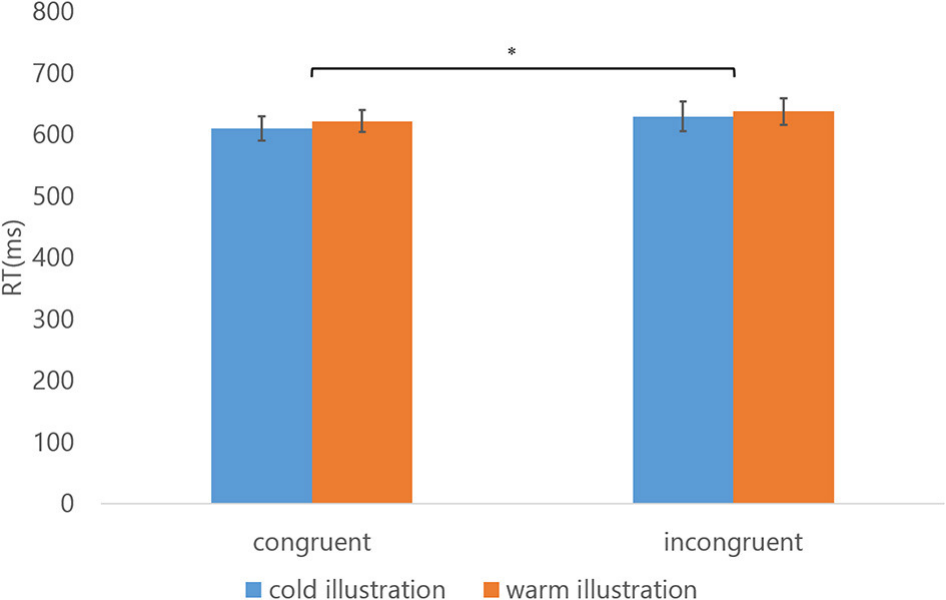
Results indicating the mean of RTs (Experiment 2). The target stimuli in two categories are indicated by the color of the bars, with blue and orange bars representing illustrations contain the meaning of coldness and warmth, respectively. The error bars show the standard errors of the mean. ^*^Indicates statistical significance of *p* < 0.05 (three-way repeated measures ANOVA).

Figure 5 shows the mean of RTs for classifying semantic meaning contained in the illustrations (“warmth” and “coldness”). A three-way repeated measures ANOVA was conducted with congruency, meaning of illustrations, and test order as factors and with RT as the dependent variable. There was a significant effect of congruency, [*F*_(1, 11)_ = 7.439, *p* = 0.020, η^2^ = 0.403]; RTs for congruent key assignments were faster than for incongruent key assignments. The difference in RTs for cold words in congruent and incongruent conditions was 20 ms (610 vs. 630 ms); for warm words was 15 ms (622 vs. 637 ms). Neither the main effects of meaning nor test order were significant, [for meaning, *F*_(1, 11)_ = 2.372, *p* = 0.152, η^2^ = 0.177; for test order, *F*_(1, 11)_ = 0.283, *p* = 0.605, η^2^ = 0.025]: RTs of “cold” illustrations did not differ from “warm” ones, and the test order of congruent/incongruent conditions in a block did not affect the results. No significant interactions were observed, [for meaning × congruency, *F*_(1, 11)_ = 0.201, *p* = 0.663, η^2^ = 0.018]; [for congruency × test order, *F*_(1, 11)_ = 3.950, *p* = 0.072, η^2^ = 0.264]; [for meaning × test order, *F*_(1, 11)_ = 0.136, *p* = 0.719, η^2^ = 0.012]; [for meaning × congruency × test order, *F*_(1, 11)_ = 1.020, *p* = 0.334, η^2^ = 0.085], and this was expected because there was no significant difference between illustrations associated with cold/warm meaning in terms of subjective thermal intensity. The estimated Bayes factor for congruency was larger than 3, [*BF*_10_ = 4.025], which indicates the current sample size was sufficient for obtaining positive evidence of the congruency effect.

## Discussion

Previous studies have revealed the interaction between physical temperature stimulation and cognition (e.g., Williams and Bargh, 2008; Ijzerman and Semin, 2009; Inagaki and Eisenberger, 2013). Of course, there is an apparent and intuitive relationship between perceptual and conceptual thermal qualities as they display a high degree of similarity, but the influence of sensory processing on the internal processing of meaning is still undetermined. The main aim of our study was to assess the association between physical warmth and coldness and corresponding semantic processing, i.e., whether the physical temperature influences the processing of meaning. We demonstrated the congruency effect, where the congruent assignment of thermal stimuli to the response key led to shorter RTs than the incongruent key assignment. Our results indicate that perceptual-semantic correspondences influenced the discrimination speed to a semantic-word/illustrative stimulus, and that the “warm sensation-warm meaning” and “cold sensation-cold meaning” congruency facilitated semantic processing. The results are the first to demonstrate an association between physical thermal stimulation and semantic representations in terms of words or illustrations using a direct performance task.

As Experiments 1 and 2 demonstrated, the congruent physical temperature stimulations facilitated the acquisition of meaning for corresponding semantic words and illustrations. It has been proposed that the internal representation is implemented via simulations in the brain's modal systems and interfaced with the external structures (Barsalou, 2010). Previous studies (Lakoff and Johnson, 1980, 1999; Singer et al., 2006; Zhong and Liljenquist, 2006; Jostmann et al., 2009) suggested that thoughts are implemented in perceptuo-motor simulations, i.e., concepts are grounded in physical experiences though conceptual metaphor, and the language and action system may interact when processing meaningful information (Pulvermüller et al., 2005). The present study investigated how external stimulation influences the process of acquiring meaning with semantic representations (words and illustrations), without any specific conceptual metaphor (e.g., “affection is warmth”), the results indicate that the bodily experiences influenced the processing of semantic meaning. The meaning of “warmth” and “coldness” were presented in semantic (words) and visual (illustrative images) stimuli in the Experiments 1 and 2, respectively. The illustrations are the alternative representations of words presumably having the same semantic meanings and could be categorized into two groups, warm and cold, like words. Although we cannot rule out the possibility that other associations may be activated by viewing the illustrations, the association between the temperature and illustration was strong enough for participants to evaluate the subjective thermal intensity of each illustration. Hence, these illustrations can be divided into “warm” and “cold” groups successfully. Therefore, the sensory experiences were expected to affect the discrimination of both words and illustrations. The participants had learnt and memorized the meanings of each image beforehand, which might explain why the overall RTs for the illustrations were shorter than for the semantic words. A previous study has indicated that visual information can affect human thermodynamics and core body temperature (Takakura et al., 2015). In another study (Ho et al., 2014b), the results indicated that visual information can affect thermal discrimination RTs, but thermal cues did not influence color discrimination responses. Halali et al. (2017) showed that cool compared to warm temperature priming (participants were primed using touch experience and by viewing pictures of landscapes) improved cognitive control performance, and such a temperature priming effect does not necessarily require a physical touch experience. Although more elaborate research is needed to confirm whether there is a bidirectional mechanism of visual processing and thermal sensation, it is also possible temperature stimulation influenced the processing of visual information (including but not necessarily limited to higher level semantic processing).

The interaction between temperature and language processing is supported by the research of the brain's neural processes. There is activity and functional connectivity in the insular cortex when thermal stimuli are processed (Peltz et al., 2011). A meta-analyses (Liakakis et al., 2011) also found effective connectivity between the insular cortex and the area of the inferior frontal gyrus (BA44, the Broca's area for language processing). Not limited to these areas, the insular cortex is broadly connected to other language centers, such as the supramarginal gyrus for language repetition, and may play a role in coordinating higher-order cognitive processing of language (Ardila et al., 2014; Oh et al., 2014). Still, more specific investigations of brain connectivity for semantic processing in language are required.

In addition to the hypothesis that the temperature-semantic congruency effect is the result of the interaction of temperature and language processing, there are other possible alternative accounts for such an effect. The internal attention toward the hands located on the thermal display that is congruent with the meaning of the stimulus might be oriented faster than on one that is incongruent with it. Recent research has demonstrated that semantic congruency can affect attention control. For instance, Mastroberardino et al. (2015) showed through fMRI analysis that crossmodal semantic congruency can affect visuo-spatial processing in an audio–visual task. Similarly, behavioral demonstrations have shown that crossmodal semantic congruency influences visuo-spatial attention. For instance, hearing a characteristic sound makes you look at visual target objects more quickly (Iordanescu et al., 2008, 2010) as the visual processing of associated objects is enhanced by sound with congruent meaning. Moreover, Iordanescu et al. (2008) also investigated the processing level of the crossmodal enhancement. The results indicated the same characteristic sounds have no influence on the name (represented by words) search of the same objects, which proved that the activation of higher level semantic representations for the concept of objects is not enhanced by hearing the related sound. The facilitative effects only occurred at the level of visual object processing, but not the semantic level.

In the present study, we hypothesized that the presentation of thermal stimuli may influence the semantic processing of words related to thermal sensation, as these words are associated with perceptual experience spontaneously. Likewise, both semantic word and illustration stimuli were used in our experiments, and the results showed that average RTs in the congruent assignment of physical temperature and meaning were faster than in the incongruent one in both experiments. This means the congruency effect occurred during the processing of semantic and visual representations of objects and concepts. Additionally, the differences between congruent and incongruent conditions were larger in Experiment 1, indicating that the temperature-semantic congruency effect in semantic words was relatively stronger than in illustrations. Since the meanings represented in the illustrations were presumably the same as the chosen words, the association between temperature and illustration was strong enough for participants to be able to evaluate the subjective thermal intensity of each illustration and distinguish them as being in the warm or cold category, although other associations might be activated by viewing the illustrations. The visual processing for the discrimination of objects is less influenced by consistent information provided through touch, as the illustrations are in iconic forms which contain the perceptual analogy explicitly, and less effort needed to discriminate the category of warmth and coldness. In contrast to previous studies on crossmodal semantic congruency effect, the present experiment investigated the semantic congruency effect by touch-visual stimulation. The association is relatively more direct (compared to audio-visual stimulation) between physical temperature stimulation (warm or cold) and words/illustrations containing the meaning of warmth or coldness, as the objects in the environment may have more than one related sound (e.g., birds sing differently), but objects related to the concept of “warm” are always “warm.” The present study did not investigate the influence of physical temperature stimulation on audio processing of words; thus, it did not determine whether the temperature-semantic effect is modality specified. Additionally, the mechanism of crossmodal interaction in touch and semantic processing remains to be determined, particularly in other haptic senses. Although one study found that semantic congruency facilitates haptic qualities of weight, firmness, and vibration (Hecht and Reiner, 2010), the bidirectional relation between physical touch and semantic processing should be verified: Is cognition of acquiring meaning embodied in physical experience? Or does attention deployment play a more important role in facilitating semantic processing?

Such an enhanced effect may be beneficial in real-life situations. Specifically regarding the effects related to temperature, studies have confirmed that the experience of physical warmth influences affective comprehension and behavior. For instance, Williams and Bargh (2008) have demonstrated that people who held warm coffee were more likely to judge an imaginary individual as friendly and warm than those who held cold coffee. The conceptual thought and language are understood via the conceptual metaphor of “affection is warm/disaffection is cold” (See also Zhong and Leonardelli, 2008; Ijzerman and Semin, 2009; Citron and Goldberg, 2014). The temperature-semantic association demonstrated in our experiments might help us to understand the basis of such conceptual metaphor.

## Author contributions

YZ, H-NH, and JW: conceived the experiments; YZ: performed the experiments, analyzed the data, and drafted the paper; H-NH and JW: provided critical revisions.

### Conflict of interest statement

The authors declare that the research was conducted in the absence of any commercial or financial relationships that could be construed as a potential conflict of interest.
